# Radiofrequency neurolysis versus surgical neurectomy for Morton’s Neuroma

**DOI:** 10.1007/s00264-026-06824-0

**Published:** 2026-05-04

**Authors:** Javier Adán-Laguna, Laura Villarroya Martínez, Germán Puyuelo Martínez, Juan Segura-Nuez, Carlos Pindado García, Carlos Martín-Hernández, Laura Ezquerra Herrando

**Affiliations:** 1 Department of Orthopaedics, Hospital Universitario Miguel Servet, Zaragoza, Spain; 2 Department of Hematology and Hemotherapy, Hospital Universitario Miguel Servet, Zaragoza, Spain

**Keywords:** Morton’s Neuroma, Radiofrequency neurolysis, Thermocoagulation, Neurectomy, Surgical treatment

## Abstract

**Abstract:**

Morton’s neuroma is one of the most common forefoot pathologies. In recent years, radiofrequency ablation has emerged as a minimally invasive alternative to surgical excision, aiming to relieve symptoms while reducing morbidity.

**Purpose:**

To compare the efficacy and safety of radiofrequency neurolysis and surgical excision for the treatment of Morton’s neuroma at our institution.

**Methods:**

A single-center, observational, retrospective, and longitudinal study was conducted including patients treated between 2012 and 2022. Clinical data from patients who underwent either surgical excision or radiofrequency ablation were analyzed. Demographic characteristics, pain intensity assessed using the Visual Analog Scale (VAS), complication rates, and reintervention rates were recorded, with a minimum follow-up of two years. Statistical analysis was performed using SPSS version 25.

**Results:**

A total of 192 procedures were identified (110 surgical excisions and 82 radiofrequency ablations). For analyses focused on demographic and baseline characteristics, repeated procedures performed in the same patient were grouped, resulting in 144 unique cases (92 excisions and 52 radiofrequency ablations). Both techniques resulted in significant pain reduction. Surgical excision achieved greater VAS improvement (− 5.57) compared with radiofrequency ablation (− 4.3). Complications were more frequent after surgical excision (13%) than after radiofrequency ablation (3.7%). In the radiofrequency group, 26% of patients required subsequent surgical excision and 33% underwent repeat ablation. The use of radiofrequency increased from 20% during 2012–2017 to 57% during 2018–2022.

**Conclusions:**

Radiofrequency ablation is a safe and effective treatment for Morton’s neuroma, although its analgesic effect appears less durable than surgical excision. Its low complication rate and outpatient applicability make it a valuable alternative, particularly in selected patients.

## Introduction

Morton’s neuroma, first described by Thomas G. Morton in 1893 [1] is a common forefoot condition characterized by intermetatarsal neuropathic pain, typically located between the third and fourth toes. Despite being referred to as a “neuroma,” it is, in fact, a degenerative thickening of the common digital plantar nerve, associated with perineural and epineural fibrosis, oedema, and loss of myelinated fibers [2].

Its aetiology is multifactorial. Repetitive microtrauma is thought to play a predominant role. The third intermetatarsal space, where branches of the medial and lateral plantar nerves converge, contains a nerve with an increased thickness and a reduced mobility, what makes this location the most frequent in Morton’s neuroma [3]. Mechanical compression beneath the deep transverse intermetatarsal ligament, and associated forefoot deformities such as hallux valgus and metatarsus primus varus, also contribute to its development [4]. Additional theories include intermetatarsal bursitis [5] and progressive vascular compromise [6] in the pathophysiology of this affection.

### Imaging diagnosis

Although the diagnosis is primarily clinical, imaging studies are commonly used to confirm and characterize the lesion. Ultrasound is the most frequently used modality due to its accessibility, low cost, and capacity for dynamic assessment. It offers a sensitivity of 91% and a specificity of 85% [7]. Morton’s neuroma typically appears as an ovoid hypoechoic mass between the metatarsal heads, and it is considered pathological when measuring over 5 mm. Magnetic resonance imaging (MRI), with a sensitivity of 90% and a specificity of 100% [7], is usually limited for complex cases or recurrences. Both imaging modalities show comparable diagnostic accuracy [8], although ultrasound has been found to be more cost-effective. Elastography may improve detection of small neuromas [9].

### Treatment

Conservative measures such as footwear modifications, orthotic devices, and corticosteroid injections, are the first line treatment, which can be effective in up to 50% of cases [10]. However, 30% recur within a year. When conservative options fail, invasive treatments are considered. Jain et al. [11] proposed a stepwise treatment algorithm in which surgery is reserved as a final step. Nevertheless, a 2004 systematic review by Thomson et al. [12] highlighted the scarcity of high-quality studies and did not support any firm recommendations. Matthews et al. [13] proposed a new Cochrane review to update the available evidence. This review has been recently published and concludes that, although multiple interventions exist for Morton's neuroma, few have been evaluated through randomized clinical trials, and the authors recommend that future studies improve their methodology.

The two primary invasive techniques are:

- Surgical Excision

This is the most commonly employed approach in refractory cases. It involves resection of the nerve through either a dorsal (more frequent) or plantar approach with a success rates of approximately 85% [11]. However, recurrence can occur in up to 10% of cases due to stump neuroma formation or incomplete resection. Excision results in localized sensory loss but it is generally well tolerated. The complication rate is low, primarily consisting of minor wound infections, and no significant difference in outcomes has been demonstrated between the two surgical approaches [14, 15].

- Radiofrequency Neurolysis

This minimally invasive technique consists of the application of electrical current to induce either continuous thermocoagulation (> 70 °C) or pulsed neuromodulation (< 50 °C). It was first introduced by Finney et al. [16] in 1989, and this technique has gained popularity over the past decade. Although no randomized controlled trials are available, clinical case series have reported success rates of approximately 80% [17, 18]. The pulsed mode selectively targets nociceptive C and Aδ fibres, resulting in less tissue damage [19]. A low complication rate, outpatient feasibility, and technical simplicity make it a valid alternative to surgical excision. Nevertheless, relevant rates of reintervention have been observed, suggesting a potentially transient instead of definitive therapeutic effect [20, 21].

## Materials and methods

An observational, descriptive, longitudinal, and retrospective study was conducted *at* a high-volume tertiary referral center. All patients who underwent intervention for Morton’s neuroma using either surgical excision or radiofrequency ablation between January 2012 and October 2022 were included, ensuring a minimum follow-up period of two years for all cases.

It is worth noting that the sample size of our study is substantially larger than that reported in most previously published studies on this topic, in which the majority of case series do not exceed 50 patients per technique [16, 17]. This larger volume makes our study relevant to compare these popular techniques. The study was approved by the Research Ethics Committee of the entity.

A priori sample size estimation was not performed, as the study included the total population of patients who met the inclusion criteria during the study period. Data were collected from the electronic medical records system. All patient information was pseudonymized and entered into a custom-built database using Microsoft Excel.

### Exclusion criteria

- Patients who underwent surgery for other forefoot conditions during the same procedure.
- Absence of postoperative follow-up data or follow-up duration less than two years.
- Severe cognitive impairment that precluded accurate pain assessment.
- Referral to another centre for surgical treatment.
- Patients treated with corticosteroid or platelet-rich plasma (PRP) injections without undergoing surgical intervention.

The following variables were recorded: age, sex, treatment technique, inclusion and surgery dates, laterality, affected interdigital space, imaging modality used (ultrasound or MRI), neuroma size (mm), outpatient status, pre- and postoperative pain (Visual Analog Scale [VAS]), reinterventions, and associated complications.

Statistical analysis was performed using SPSS version 25 (IBM Corp., Armonk, NY). Statistical comparisons were performed using Student’s *t* test, Pearson’s chi-square test, Pearson’s correlation coefficient, and Phi and Cramer’s V coefficients, as appropriate.

The surgical techniques were standardized across all cases, with minor variations depending on the primary surgeon. Both techniques are described below:

- Surgical Excision

Using locoregional anaesthesia and sedation, with the patient in the supine position and a thigh tourniquet applied, a dorsal approach was performed. A longitudinal incision approximately 3 cm in length was made over the affected interdigital space. Careful dissection exposed the deep transverse intermetatarsal ligament, which was systematically sectioned to allow visualization of the neuroma. The nerve was resected as proximally as possible, with distal sections in both digital branches. The wound was then closed in layers, and a compressive dressing was applied. Postoperative management included immediate weight-bearing with a rigid-soled shoe, oral analgesics as needed, and antithrombotic prophylaxis for 20 days.

- Radiofrequency Neurolysis

A pulsed radiofrequency generator (model TLG-10) was used, along with a 23G–60 mm cannula with a 5-mm active tip. Under local anaesthesia and ultrasound or anatomical guidance, the needle was inserted into the affected interdigital space. A sensory stimulation (50 Hz, up to 5 V) was applied to confirm the precise location of the painful point. A single 4-min cycle of pulsed radiofrequency (1 Hz, 45 V) was then administered. The procedure was performed on an outpatient basis, and immediate postoperative weight bearing was allowed.

## Results

A total of 334 surgical procedures with a diagnosis of Morton’s neuroma were identified between January 2012 and October 2022. After applying the exclusion criteria, 192 valid procedures were included in the analysis.

For comparative analysis between treatment techniques, only the first intervention per patient was considered, resulting in a final sample of 144 patients: 92 initially treated with surgical excision (64%) and 52 with radiofrequency neurolysis (36%).

**Fig. 1:**
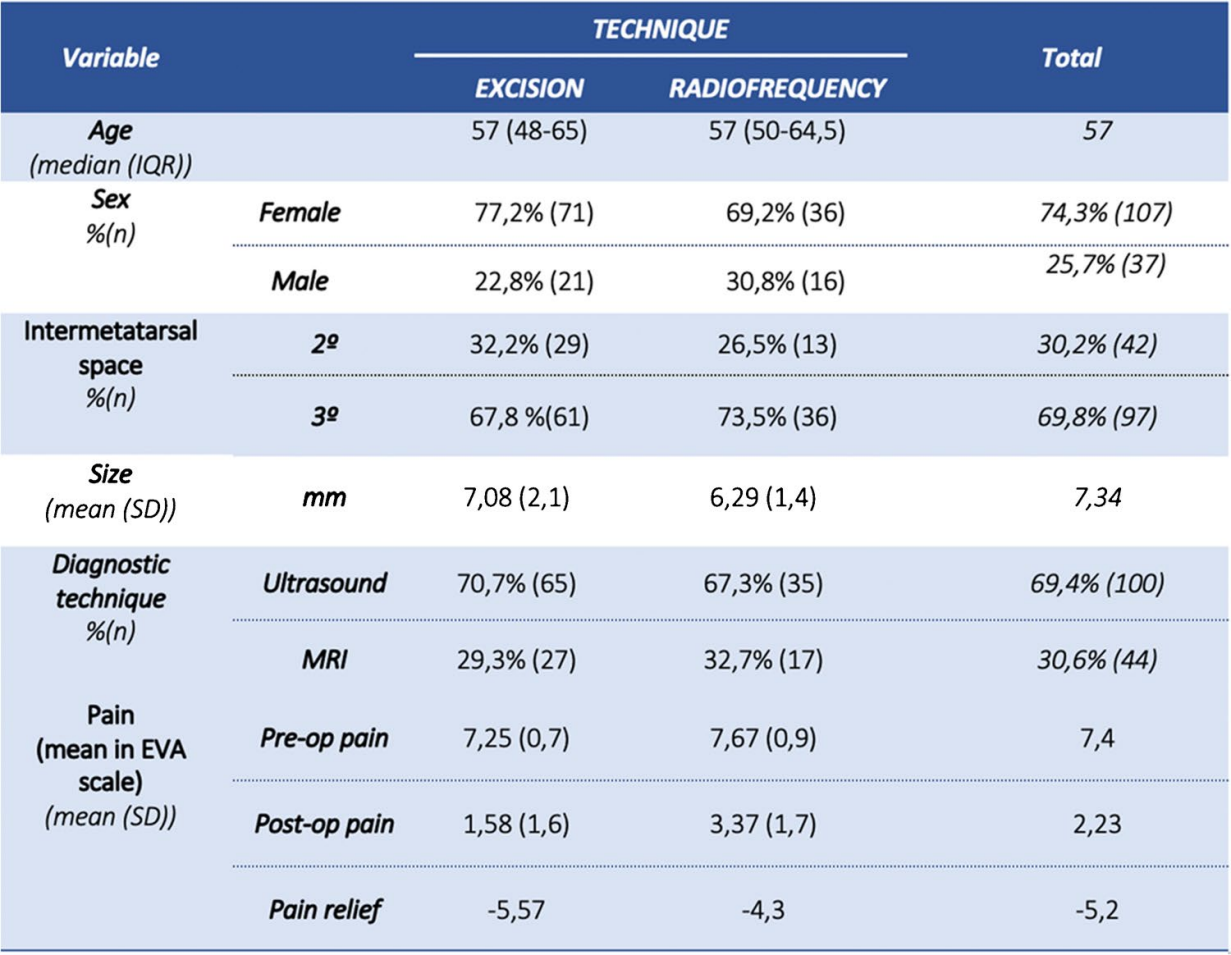
Demographic data of the sample

Demographic characteristics of the sample are summarized in Fig. 1. The mean age was 56 years in the excision group and 57 years in the radiofrequency group, with no statistically significant differences. Female patients accounted for 74.3% of the total sample. The third interdigital space was the most frequently affected location in both groups (approximately 70%), followed by the second space. The left foot was involved in 57.7% of cases.

Regarding neuroma size, statistically significant differences were observed between the two groups: the mean diameter was 6.29 mm in patients treated with radiofrequency and 7.08 mm in those who underwent surgical excision. Although a statistically significant difference in neuroma size was observed between groups, the absolute difference was less than 1 mm.

During the study period, the use of radiofrequency neurolysis showed a marked increase. While it accounted for only 20% of procedures between 2012 and 2017, it rose to 57% between 2018 and 2022, becoming a commonly used alternative to open surgery.

Both techniques achieved a significant reduction in pain, as measured using the Visual Analog Scale (VAS). The reduction was greater in the excision group (−5.57) compared to the radiofrequency group (−4.3), a difference that was statistically significant.

**Fig. 2:**
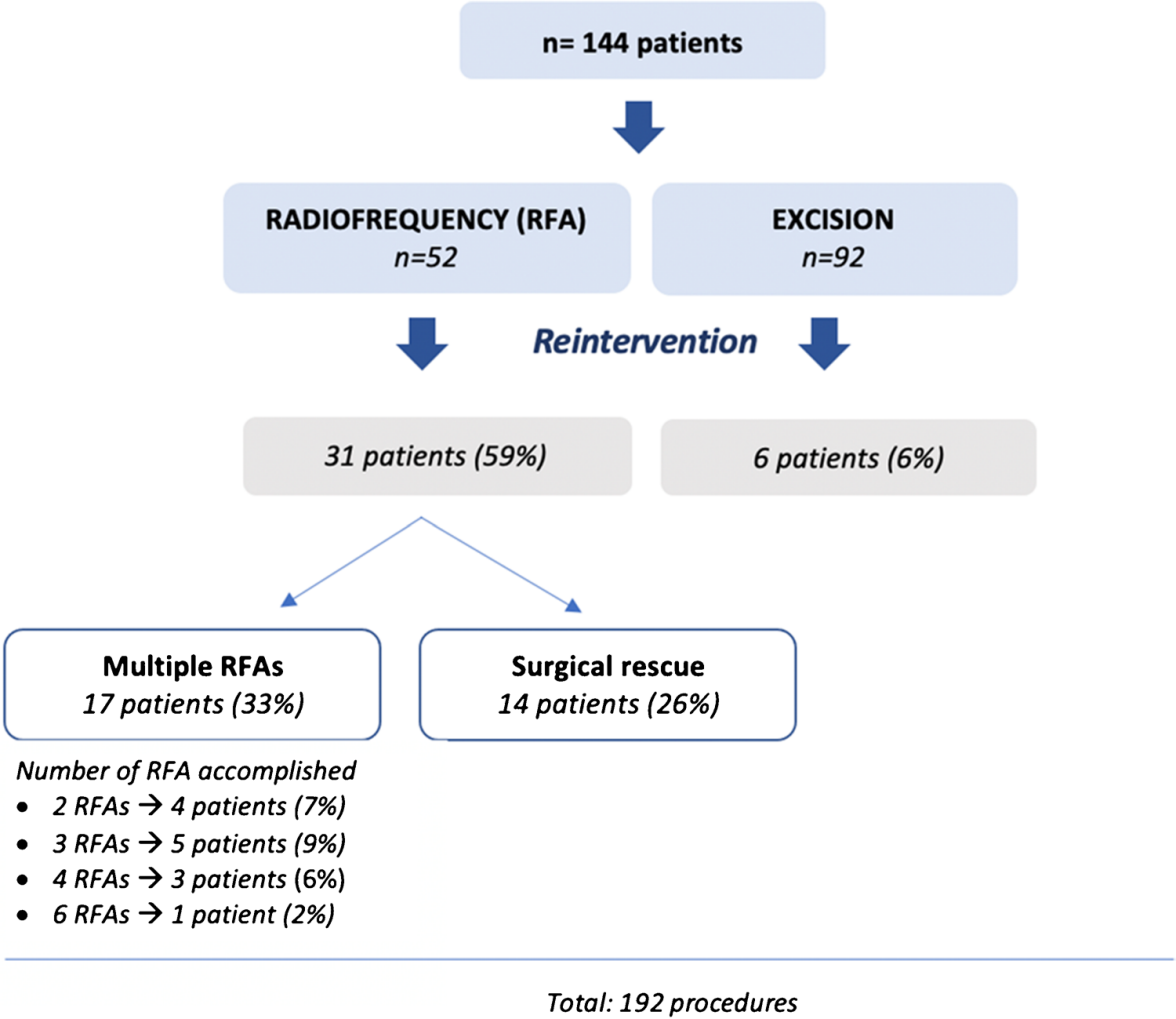
Analysis of reintervention

Figure 2 shows the distribution of patients who required reintervention. The reintervention rate was 6% in the excision group versus 59% in the radiofrequency group. Among patients treated with radiofrequency, 33% required repeated ablation sessions, and 26% underwent rescue surgery. However, it is important to point out that 74% of patients initially treated with radiofrequency avoided open surgery, supporting its role as a valuable first-line option in selected clinical profiles.

Regarding the imaging modality used for diagnosis, no significant differences were found in reintervention rates between patients diagnosed by ultrasound and those diagnosed by MRI, as illustrated in Table 1.

**Table 1:**
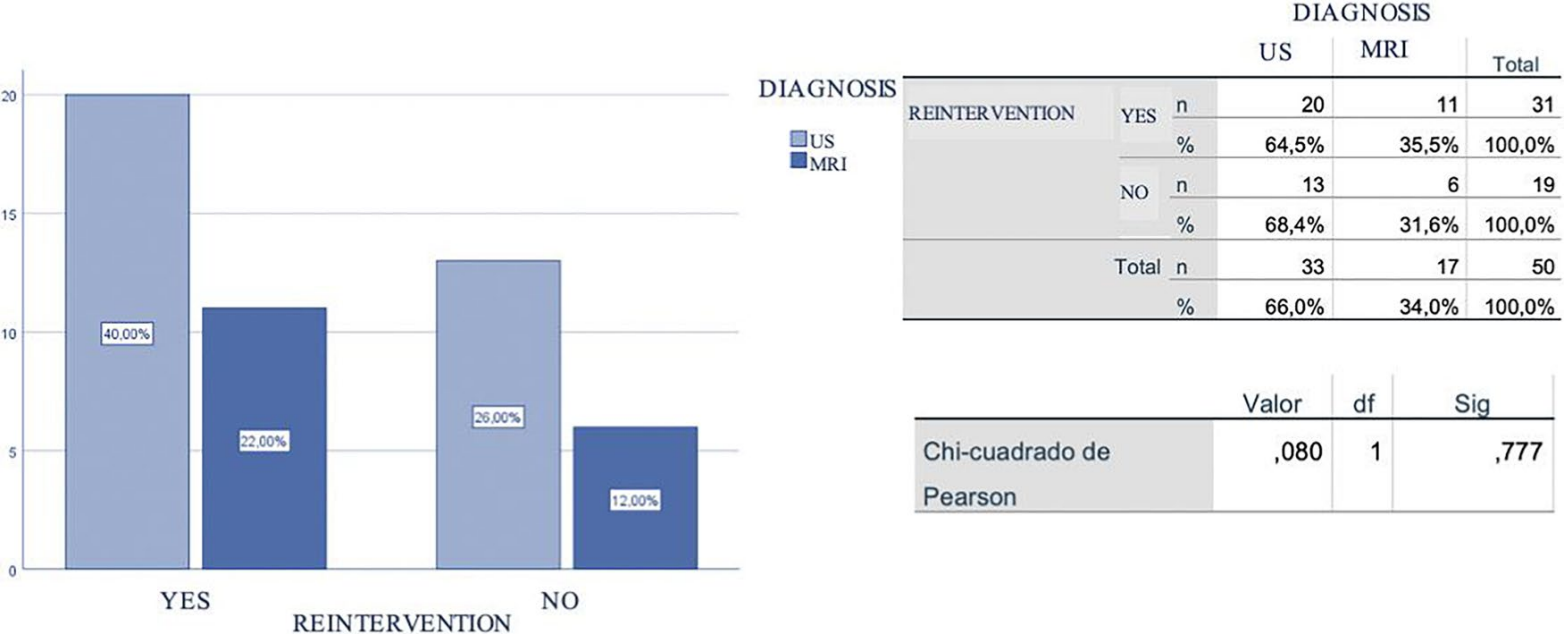
Comparison between reintervention and diagnostic technique

Table 2 analyzes the relationship between intervention date and reintervention rate. No progressive improvement was observed in radiofrequency outcomes over time, suggesting consistent efficacy despite increased experience with this technique. Similarly, no significant differences were found when analyzing the reintervention rate according to the neuroma size at the time of diagnosis (Table 3)*.*

**Table 2:**
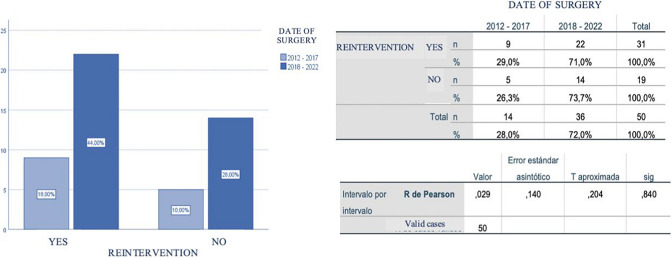
Comparison between reintervention and date of surgery

**Table 3:**
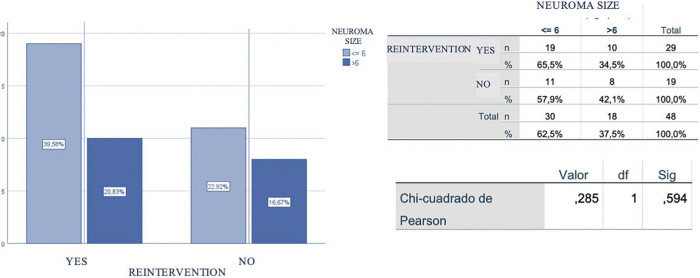
Comparison between reintervention and size of neuroma

**Fig. 3:**
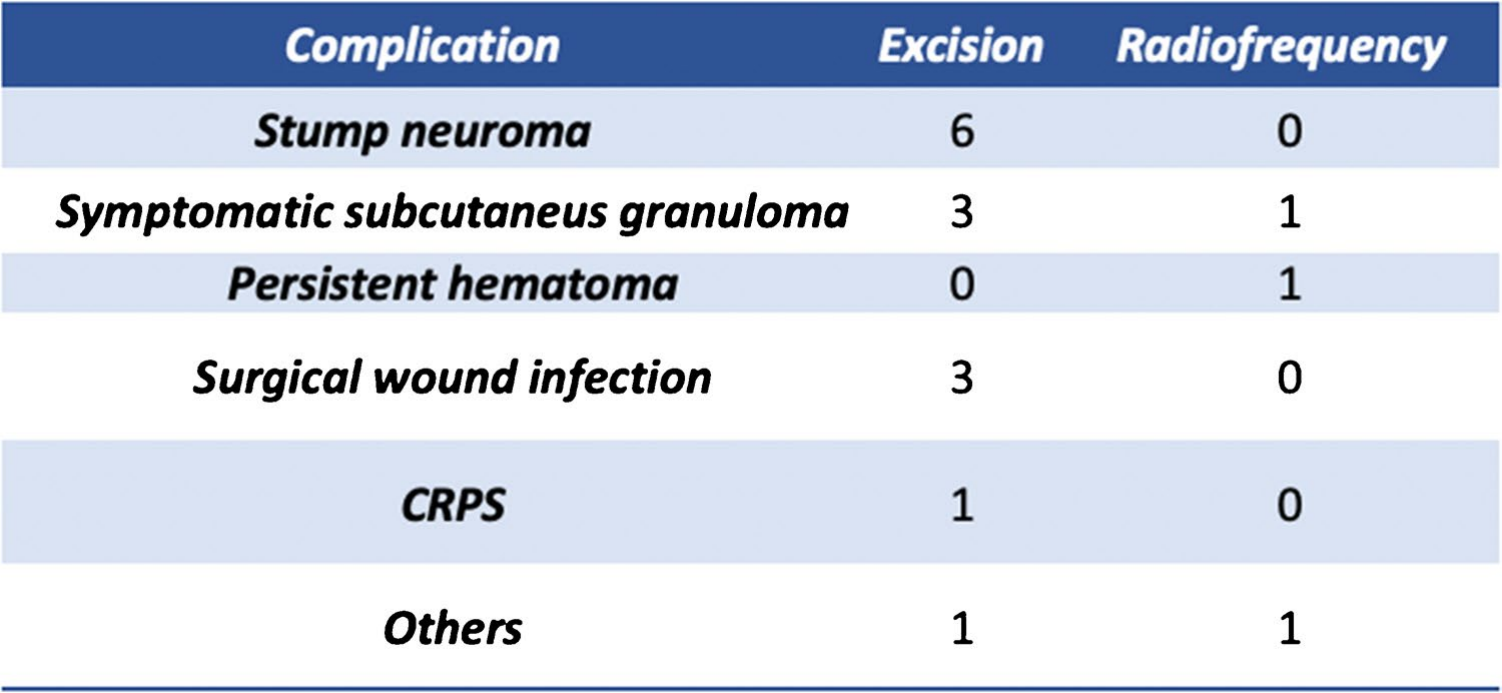
Complications suffered during follow-up

Finally, the overall complication rate was 9%, with a significant difference between groups: 13% in the surgical excision group compared to 3.7% in the radiofrequency group, as shown in Fig. 3, most complications were mild, such as superficial wound infections. One case of complex regional pain syndrome was reported after surgery, which evolved favorably with rehabilitative treatment.

## Discussion

The results obtained in our series suggest that both surgical excision and radiofrequency neurolysis are effective techniques for the treatment of Morton’s neuroma. However, they differ in terms of symptom relief, reintervention rates, and complication profiles.

One of the most relevant findings of the study is that no significant correlation was found between neuroma size and the intensity of pain reported by patients. This result, consistent with the findings of Bencardino et al. [22] and Sharp et al. [23], supports the hypothesis that small lesions may be highly symptomatic, while larger neuromas can be silent. In our series, some patients with neuromas as small as 4 mm reported high-intensity pain (VAS ≥ 7), whereas others with neuromas larger than 10 mm presented with only moderate symptoms. This suggests that size alone should not determine the therapeutic approach.

It was also observed that patients with higher preoperative VAS scores experienced greater pain reduction following intervention. This pattern, aligned with the findings of Genon et al. [24] and Lu et al. [25], suggests that higher baseline pain intensity may be associated with a more complete therapeutic response.

Regarding diagnostic imaging, both ultrasound and magnetic resonance imaging proved to be useful, with no significant differences in reintervention rates between them. These findings are consistent with prior reviews [7, 8], which emphasize the high sensitivity and specificity of both techniques, although ultrasound has the advantage of lower cost and greater accessibility. Regarding neuroma size, statistically significant differences were observed between the two groups, with a mean diameter of 6.29 mm in patients treated with radiofrequency and 7.08 mm in those who underwent surgical excision. However, the absolute difference was less than 1 mm. Given that ultrasound is an inherently operator-dependent technique, a discrepancy of this magnitude should be interpreted with caution, as it may reflect measurement variability rather than a true difference between groups. Accordingly, the clinical relevance of this finding appears limited.

The significantly higher reintervention rate observed in the radiofrequency group (59% vs 6% after surgical excision) suggests that, while thermocoagulation may offer effective short-term symptom relief, its durability as a single definitive treatment is limited in a considerable proportion of patients. In contrast, surgical excision remains the reference standard, with consistently favorable outcomes reported in the literature, although not without a small risk of persistent or recurrent pain in 8% of patients [26].

Published studies on radiofrequency ablation have generally reported good outcomes, but these findings are based on small and heterogeneous series. Brooks et al. demonstrated improved efficacy with up to three treatment cycles, suggesting that repeated applications may enhance outcomes [20], while Connors et al. described high patient satisfaction at mid-term follow-up with low progression to surgery [27]. However, our higher reintervention rate may reflect differences in patient selection, real-world practice conditions, or the inclusion of more resistant neuromas.

Importantly, despite the need for additional procedures, 74% of patients initially treated with radiofrequency avoided surgical excision. This supports its role as a minimally invasive, surgery-sparing option in selected cases. Therefore, radiofrequency may be best considered within a staged treatment strategy rather than as a direct alternative to neurectomy, with patients appropriately counselled regarding the potential need for repeat interventions.

In terms of safety, radiofrequency showed a significantly lower complication rate (3.7%) compared to surgery (13%). Complications associated with excision were primarily superficial infections and stump neuroma formation, while adverse events in the radiofrequency group were minimal and transient. This favourable safety profile, combined with the possibility of outpatient management, makes radiofrequency an attractive alternative from both economic and logistical point of view.

Our study presents several limitations inherent to its retrospective observational design. The primary limitation lies in the reliance on clinical records, which may affect data consistency. In addition, although the technique was standardized, procedures were performed by different surgeons over the years, potentially introducing operator bias. Moreover, pain assessment was conducted using the Visual Analog Scale (VAS), which is useful for short-term follow-up but limited in capturing the full patient experience. It should also be noted that follow-up duration was shorter for more recent procedures, potentially leading to an underestimation of reintervention rates. Nevertheless, a minimum two-year follow-up is considered enough for consistent results.

This study has the inherent limitations of a non-randomized design, including the potential for selection bias and residual confounding. Although a statistically significant difference in neuroma size was observed between groups, the absolute difference was less than 1 mm. Given that these measurements were obtained with ultrasound—an inherently operator-dependent technique—such a small discrepancy is unlikely to be clinically meaningful and should be interpreted with caution. Importantly, the remaining baseline characteristics of the cohort were comparable between groups, which supports the overall validity of the comparisons while acknowledging the need for cautious interpretation of the findings.

Despite these limitations, our study represents one of the largest series published to date comparing these two techniques and provides relevant data on their relative efficacy and safety profiles.

## Conclusions

The present study supports that there is no direct relationship between the size of Morton’s neuroma and the pain perceived by the patient. Small neuromas may cause intense pain, while larger lesions may be clinically insignificant. This finding has important implications for treatment decision-making, as it underlines the need to prioritize clinical presentation over radiological dimensions.

Pulsed radiofrequency is a useful technique for reducing pain, particularly in selected patients. Although symptom relief is generally less than with surgical excision, the possibility of avoiding surgery in a high percentage of patients (74% in our sample) supports its use as a first-line treatment in cases with moderate pain.

Despite the higher need for reintervention following radiofrequency treatment, its safety seems to offer better outcomes, with a minimal complication rate and only mild, self-limiting side effects. This makes it an attractive option for outpatient use.

The results of this study suggest that both radiofrequency and surgical excision are valid therapeutic strategies. The choice between them should be individualized based on the patient’s clinical characteristics, risk profile, and informed preference.

Finally, our findings highlight the need for well-designed randomized clinical trials to compare both techniques with a higher level of evidence, to help in the development of more accurate therapeutic protocols.

## Data Availability

Data are available upon reasonable request.
